# Chemical Profile and Biological Potential of Non-Polar Fractions from *Centroceras clavulatum* (C. Agardh) Montagne (Ceramiales, Rhodophyta)

**DOI:** 10.3390/molecules16087105

**Published:** 2011-08-19

**Authors:** Otávio P. Rocha, Rafael De Felício, Ana Helena B. Rodrigues, Daniela L. Ambrósio, Regina Maria B. Cicarelli, Sérgio De Albuquerque, Maria Claudia M. Young, Nair S. Yokoya, Hosana M. Debonsi

**Affiliations:** 1 Departamento de Física e Química, Faculdade de Ciências Farmacêuticas de Ribeirão Preto – USP, Av. do Café, s/n, CEP 14040-903, Monte Alegre, Ribeirão Preto-SP, Brazil; 2 Faculdade de Ciências Farmacêuticas de Araraquara, Departamento de Ciências Biológicas – UNESP, Rodovia Araraquara-Jaú, Km 01. CEP 14801-902, Araraquara-SP, Brazil; 3 Departamento de Análises Clínicas, Toxicológicas e Bromatológicas, Faculdade de Ciências Farmacêuticas de Ribeirão Preto – USP, Av. do Café, s/n, CEP 14040-903, Monte Alegre, Ribeirão Preto-SP, Brazil; 4 Núcleo de Pesquisas em Fisiologia e Bioquímica Vegetal, Instituto de Botânica, Secretaria de Estado do Meio Ambiente de São Paulo, Av. Miguel Estefano, 3687, Água Funda, CEP 04301-012, São Paulo-SP, Brazil; 5 Núcleo de Pesquisas em Ficologia, Instituto de Botânica, Secretaria de Estado do Meio Ambiente de São Paulo, Av. Miguel Estefano, 3687, Água Funda, CEP 04301-012, São Paulo-SP, Brazil

**Keywords:** *Centroceras clavulatum*, GC-MS analysis, Rhodophyta, trypanocidal activity, antifungal activity

## Abstract

The present study reports the Gas Chromatography-Mass Spectrometry (GC-MS) evaluation of the hexanes and dichloromethane fractions from extracts of the red alga *Centroceras clavulatum* (C. Agardh) Montagne. Twenty three compounds were identified, totaling ca. 42% of both fractions (0.18 g mass extract). The main constituents of the fractions were hexadecanoic acid (17.6%) and pentadecanoic acid (15.9%). Several secondary metabolites with interesting biological activity, such as (-)-loliolide, neophytadiene, phytol were identified. In addition, several classes of secondary metabolites, including phenolic compounds (e.g., phenylacetic acid), terpene derivatives, fatty acids, halogenated compound (e.g., 2-chlorocyclohexenol), lignoids, steroids, esters, amides (e.g., hexadecanamide), ketones, carboxylic acids, aldehydes and alcohols were observed. The occurrence of several of these structural classes is described for the first time in this species. The same fractions analyzed by GC-MS, and a separate set of polar fractions, were evaluated against two life cycle stages (epimastigote and trypomastigote forms) of the protozoan *Trypanosoma cruzi* and against phytopatogenic fungi *Cladosporium cladosporiodes* and *C. sphaerospermum*. The dichloromethane fraction was active against both *T. cruzi* forms (epimastigote IC_50_ = 19.1 μg.mL^−1^ and trypomastigote IC_50_ = 76.2 μg.mL^−1^). The hexanes and ethyl acetate fractions also displayed activity against both fungi species (200 μg) by TLC-bioautography.

## 1. Introduction

The family Ceramiaceae (Ceramiales, Rhodophyta) consists of more than one hundred genera, and 28 of them are found along the Brazilian coast [1,2], including the type genus *Ceramium* Roth, and *Centroceras* Kützing. *Centroceras clavulatum* (C. Agardh) Montagne has a wide distribution and common occurrence around the World, and it is the only species of the genus reported in Brazil [3]. 

Recent reports have shown biological activity in red algae. An interesting antifouling property, for example, was demonstrated by *Ceramium botryocarpum* A.W.Griffiths ex Harvey, which has been used in France in aquaculture tanks to limit the growth of invasive green algae (*Cladophora*, *Bryopsis* and *Ulva* genus) [4]. 

Hayee-Memenon and co-workers identified secondary metabolites of *Centroceras clavulatum* from Karachi (Pakistan) by GC-MS analysis; fatty acids and sterols were detected [5]. The present study reports the identification of volatile compounds of *C. clavulatum* collected from the coast of São Paulo, in southeastern Brazil, in order to investigate chemical profile of Brazilian *C. clavulatum*. In addition, this research was encouraging since essential fatty acids are precursors of prostaglandins, which are becoming increasingly important to the pharmaceutical industry [6].

The biological potential demonstrated by marine red algae [7,8,9], suggests that additional analysis to evaluate the trypanocidal activity would be worthwhile. Activity in this study was assessed against the flagellated protozoan *Trypanosoma cruzi* [10], the causative agent of Chagas’ disease, which affects 16-18 million people around the World and more than 100 million live in endemic areas, making it one of the major neglected tropical diseases [11,12,13]. Currently, between 12 and 14 million people are infected by *T. cruzi*. Also, one of the routes of transmission of infection by *T. cruzi* is by the placenta, resulting in the emigration of large numbers of infected women from rural areas to cities where there are no vectors of transmission [14]. Organ transplantation is another source of transmission. This mechanism of transmission may trigger the acute phase of the disease, since the individuals who receive transplanted organs take immunosuppressive drugs, and consequently are less resistant to infection [15]. There is no vaccine available, and few effective treatments. Benznidazole (Roche) is the most commonly drug used in Brazil. The identification of new chemotherapeutic methods and/or vaccines is the greatest challenge for controlling Chagas’ disease. The increase of the use of natural products for medicinal purposes has caused renewed interest in understanding the characteristics of substances with trypanocidal activity.

Antiparasitic activity against *Leishmania* and *Trypanosoma* genera has been described in extracts and compounds isolated from marine organisms and red algae [16,17]. Some results using extracts of seaweed such as *Fucus evanescense* and *Pelvetia babingtonii* showed a potential inhibition on DHOD (dihydroorotate dehydrogenase), an essential enzyme in *T. cruzi* [10]. Trypanocidal and antifungal activities of some non-polar fractions were also reported in the marine red alga *Bostrychia tenella* (J.V. Lamouroux) J. Agardh [9] showing high trypanocidal potential for this species. 

In this way, the present work reports the chemical constituents of non-polar fractions from *Centroceras clavulatum*, as well as its biological activity against two life cycle stages (epimastigote and trypomastigote forms) of *Trypanosoma cruzi*. Also, considering the availability of red algae species in Brazil, our biological studies are directed towards the discovery of new antifungal agents based on a simple bioautographic assay. Thus, the fractions of differing polarity from *C. clavulatum* were selected aiming to discovery any antifungal potential against the phytopatogenic fungi *Cladosporium sphaerospermum* and *C. cladosporioides* [18].

## 2. Results

### 2.1. Chemical Composition of CC-H and CC-D Fractions

Twenty-three compounds were identified, totaling ca. 42% of the fractions (0.18 g from crude extract; Table 1). The main constituents of the fractions were hexadecanoic acid (17.6%) and pentadecanoic acid (15.9%), secondary metabolites interesting for their biological activity and/or structural diversity such as neophytadiene, phytol, (-)-loliolide, phenolic compounds (e.g., phenylacetic acid), terpene derivatives, alkaloids, fatty acids, halogenated compounds (e.g., 2-chlorocyclohexenol), lignoids, steroids, esters, amides (e.g., hexadecanamide), ketones, carboxylic acids, aldehydes and alcohols. Since the only alkaloid described in the literature for the *C. clavulatum* is α-kainic acid [19], the present study describes the occurrence of these alkaloids for the first time for this species.

### 2.2. Trypanocidal and Antifungal Activities of CC-H and CC-D Fractions

The dichloromethane fraction was active against the two forms of *Trypanosoma cruzi*, epimastigote IC_50_ = 19.1 μg/mL and trypomastigote IC_50_ = 76.2 μg/mL, respectively, and the hexane fraction also showed interesting antifungal activity against *Cladosporium cladosporiodes* and *C. sphaerospermum* (Table 2). 

**Table 1:** Identification of compounds from hexanes and dichloromethane fractions from *Centroceras clavulatum* (Ceramiales, Rhodophyta).

Compound	Peak area total ± (%)	% Match withthe data bank
2-Chlorocyclohexenol	0.1	93
Phenylacetic acid	0.1	91
Hexahydro-2*H*-azepin-2-one	0.1	94
Tetradecanoic acid *	1.6	92
Octadecanoic acid	0.1	91
6,10,14-Trimethyl-2-pentadecanone	0.5	94
3-Hydroxy-β-ionone-5,6-epoxide	0.1	94
1,6,6-Trimethyl-7-(3-oxo-but-1-enyl)-3,8- dioxatricyclo[5.1.0.0 2,4]-octan-5-one	0.1	90
Eicosanoic acid	1.1	90
Hexadecanoic acid *	17.6	94
Pentadecanoic acid *	15.9	92
(*Z*)-9-Octadecenoic acid	1.7	91
(-)-Loliolide	0.5	92
Neophytadiene	0.5	95
Phytol	0.5	94
1-Octadecanol	0.3	96
Dihydro-5-tetradecyl-2(3*H*)-furanone *	0.6	90
Hexadecanamide	0.1	94
Tetradecanamide	0.1	91
*N*-Tetradecanoic acid amide	0.1	90
Dodecanamide	0.1	90
(Octyloxy)-benzene **	0.1	91
3β-Cholest-5-en-3-ol	0.1	91
Cholesta-3,5-dien-7-one *	0.5	95
Cholest-4-en-3-one	0.1	93

* Compounds identified in hexane and dichloromethane fractions; ** Compound identified only in the dichloromethane fraction; ± The % is related to total amount found in both fractions.

**Table 2:** Trypanocidal and antifungal activities of fractions from *Centroceras clavulatum* (Ceramiales, Rhodophyta).

Fractions	Cladosporium cladosporioides * R_f_ (potential)	Cladosporium sphaerospermum * Rf (potential)	*Trypanosoma cruzi* (IC_50_, μg/mL)	
			Epimastigote form **	Trypomastigote form ***
CC-H	Origin to 0.84 ( **3**)	Origin to 0.84 ( **3**)	**21.7**	858.7
CC-D	(i)	(i)	**19.1**	76.2
CC-M	0.40 (1)	(i)	91.1	18.2
CC-A	0.27 (2); 0.38 (3); 0.52 (3)	0.27 to 0.61 (1)	122.1	39.8

* Antifungal activity: R_f_ and intensify between parenthesis according legend: i – inactive; 1 – weak activity; 2 – moderate activity; 3 – strong activity; *** Trypanocidal activity: IC_50_ in μg/mL. Control gentian violet 31 μg/mL (trypomastigotes). benznidazol 9.01 μg/mL (epimastigotes).

## 3. Discussion

### 3.1. Chemical Composition of CC-H and CC-D Fractions

The highlighted technique, GC-MS, is a useful tool in modern food, medicine and biological research for the separation and identification of components of organic mixtures, and this method has already been applied successfully for the analysis of terpenoids, especially mono- and sesquiterpenes in various essential oils [20]. In addition, the trypanocidal activity of the dichromethane and hexane fractions, IC_50_ of 19.1 and 21.7 μg/mL, respectively for epimastigote forms; as well as the methanol extract, IC_50_ of 18.2 μg/mL (trypomastigote forms) was more potent than the positive control, gentian violet (IC_50_ of 31 μg/mL), so the non-polar fractions were selected for the chromatographic separation in order to obtain pure substances. The GC-MS analysis of the red alga, *Centroceras clavulatum* led to the identification of 23 major components (Table 1), accounting for ~42% of the total components present. 

The major identified components of CC-H and CC-D fractions of *C. clavulatum* were fatty acids, specifically tetradecanoic acid (1.6%), pentadecanoic acid (15.9%), hexadecanoic acid (17.6%), eicosanoic acid (1.1%), and the unsaturated fatty acid (*Z*)-9-octadecenoic acid (1.7%) (Table 1). This result is in accordance with previous reports about fatty acids detected in Rhodophyta [6,10,21]. The total fatty acid composition of nine species of Rhodophyta showed a characteristic distribution, with high relative levels of the acids 16:0 (hexadecanoic acid), 18:1(n-7), 18:1(n-9), 20:5(n-3) and 20:4(n-6) [21]. The GC-MS results obtained in this study however were significantly different from the one previously reported by Hayee-Memenon and coworkers [6], mainly due to their methodology. In their experiments, the crude extract of *C. clavulatum* was chemically treated with alkali and acid solutions, and the organic fractions were submitted to methylation in order to promote esterification of free fatty acids. Based on this methodology, the major compounds of fatty acids from *C. clavulatum* were methyl tetradecanoate, methyl pentadecanoate, methyl hexadecanoate and methyl heptadecanoate. In addition, this previous study identified some unsaturated fatty acids and different sterols. On the other hand, we identified other classes of chemical compounds, showing the great metabolic diversity this species is able to synthesize. Also, this study highlights the need for future chemical investigation of this species, since many compounds, like amides, are known to possess a large range of biological activities that was not evaluated in this study. The differences in the GC-MS profiles of *C. clavulatum* collected from different locations could be explained by the fact that plants often produce different amounts of phycochemicals when are growing in different geographical regions [22]. 

Except for the five major components of the analyzed fractions, others secondary metabolites identified in *C. clavulatum* were found in a less than 1.00%. The substance (-)-loliolide is a bicyclic lactone commonly found in marine algae [23]. The three identified sterols 3β-cholest-5-en-3-ol, cholesta-3,5-dien-7-one and cholest-4-en-3-one; the cholesterol (3β-cholest-5-en-3-ol) is a steroid with a wide occurrence in marine organisms, including algae. Analysis involving different classes of algae showed that steroid profile has been considered an important parameter to establish chemitaxonomic correlations [24]. Cholesta-3,5-dien-7-one and cholest-4-en-3-one, which are less common than cholesterol, have been previously reported in the marine red alga *Bostrychia tenella* [10]. 

### 3.2. Biological Activities of CC-H and CC-D Fractions

The CC-H fraction of *Centroceras clavulatum* showed activity against the epimastigote form and it was inactive against the trypomastigote form of *Trypanosoma cruzi*. However, the CC-D fraction was active against both *T. cruzi* forms. In both fractions, fatty acids were present, but not necessarily in equal proportion. The antifungal activities of fractions against phytopatogenic fungi *Cladosporium cladosporioides* and *Cladosporium sphaerospermum* were also evaluated in this work, and the hexanes fraction showed activity in different levels of lethality (Table 2). This result suggests the selectivity of some compounds or the synergism of them against the pathological agent. Our results can be related to some antibacterial and antifungal properties of algal extracts which contain saturated and unsaturated fatty acids [25,26]. Furthermore, mixtures well-defined among fatty acids of up to 22 carbons could improve the penetration of herbicides, contributing indirectly as antifungal agents [27]. 

## 4. Experimental

### 4.1. Algal Collection

Specimens of *Centroceras clavulatum* were collected in June 2007 on the rocky shore of Brava Beach, Ubatuba, São Paulo State, Brazil (23°30’10.8”S; 45°10’21.5”W). The algal material was identified, washed in seawater, cleaned, and maintained at a cold temperature (4 °C). Voucher specimens were deposited at Herbário do Instituto de Botânica “Maria Eneyda P. Kauffmann Fidalgo”- SP, Brazil, with the accession number CCIBt 391090.

### 4.2. Extraction and Chromatographic Separation

The dried and cleaned algae were powdered (120 g) and extracted by maceration three times using CH_2_Cl_2_-MeOH (2:1 v/v) at room temperature. The solvent was evaporated under reduced pressure, and the obtained crude extract (3.24 g) was partitioned with organic solvents, yielding the hexanes [CC-H] (0.29 g), dichloromethane [CC-D] (0.14 g), ethyl acetate [CC-A] (0.10 g) and methanol [CC-M] (0.73 g) fractions, besides the resulting salt residue. In this study, the CC-H and CC-D fractions were analyzed by GC-MS in order to identify non-polar metabolites and evaluate their biological activities. In addition, all the fractions were evaluated by various bioassays to determine the potential activity of this algal species.

### 4.3. GC-MS Analysis

GC-MS analyses were performed on a Shimadzu QP 2010 instrument using a DB-17MS column [30 m × 0.25 mm i.d, 0.25 μm film thickness]. Operating conditions were as follows: helium as the carrier gas with a flow rate of 1.49 mL/min; column temperature 80 °C for 6min then increasing at 24.4 °C /min from 80 to 300 °C; injector temperature, 260 °C; volume injected, 1 μL; split ratio, 20.0. MS were recorded in electron ionization (EI) mode, with energy of 70 eV. The ion source temperature was 200 °C; 3.00 min solvent cut time. The compounds were identified by comparison with the data in the Wiley 7.0 and NIST libraries.

### 4.4. Biological Evaluation

#### 4.4.1. Trypanocidal Activity (Epimastigote Form)

Epimastigote forms of *Trypanosoma cruzi* (Y strain) were maintained in liver-infusion tryptose medium (LIT), supplemented with 10% fetal calf serum [28]. For the *in vitro* assay, the parasites (log phase) were diluted to 10^5^ parasite/mL and 100 μL of the suspension was distributed in 96-wells plate. The fractions CC-H and CC-D were dissolved in DMSO, added in different concentrations to the wells and the plate was incubated in humid chamber for 72h at 28 °C. In the negative control was added the same amount of DMSO. All assays were performed in triplicate. The trypanosomes were counted using the Neubauer chamber and the percentage of dead parasites was calculated based on the positive control. The activities of the compounds were expressed as IC_50_ values, corresponding to the concentration that causes death of 50% of the parasites. The IC_50_ of benznidazole was determined by the same methodology.

#### 4.4.2. Trypanocidal Activity (Trypomastigote Form)

The CC-H and CC-D fractions were evaluated *in vitro* against trypomastigote blood forms of *Trypanosoma cruzi* (Y strain), which were obtained from parasitic culture in LLCMK2 cells [29]. The cells were cultivated in RPMI 1640 medium, supplemented with 2 nM L-glutamine, 10 nM NaHCO_3_, 100 U/mL of penicillin, 100 μg/mL of streptomycin and 5% inactivated fetal bovine serum, in culture bottles at 37 °C in an atmosphere with 5% CO_2_, and 95% humidity. Infected blood with trypomastigote forms was added to the culture, and the mixture was incubated for 24 hours. Following this initial treatment, cells were washed with culture medium, only leaving the parasite-infected cells. After about 5 days, when the process of cellular lysis had started and the trypomastigote forms in the medium were observed, the supernatant containing trypomastigote forms was removed, parasite concentration was determined and measured (10^6^ parasites/mL) into 96-well microplates. Samples fractions of *C. clavulatum* in concentrations of 0.5, 2.0, 8.0 and 32.0 μg/mL were added to the mixtures. 

After 24 hours of incubation, the biological activity was determined using a colorimetric assay with 3-(4,5-dimethylthiazol-2-yl)-2,5-diphenyltetrazolium bromide (MTT, ACROS Organics). First MTT (10 μL, 5 mg/mL in each plate) was added and incubated at 37 °C for 4 hours. Subsequently, acidified isopropanol [100 μL of 0.04 N HCl (aq) in isopropanol] was added to the solutions, and the microplate absorbances were measured in a Sunrise – Tecan apparatus with a 570 nm filter (reference at 630 nm). The data were processed by the program Magelan 3 (TECAN). A DMSO solution was used as the negative control with the same concentrations as the evaluated samples. The activity of the positive control (benznidazol) was verified quantitatively using a previously described methodology [30]. The fractions were prepared in triplicate and measured for their trypanocidal activity, as expressed by the percentage of activity (%AE) according to the formula:

%AE = [(AE-AEB)/(AC-ACB)] × 100

where AE represents the “absorbance of tested plates,” AEB is the “absorbance of plates containing medium and sample,” AC is the “absorbance of plates containing negative control,” and ACB is the “absorbance of plates containing culture medium.” All the IC_50_ values were calculated by nonlinear regression equation, using the computer program GraphPad Prism v.4.02.

#### 4.4.3. Antifungal Activity

The phytopathogenic microorganisms used in the antifungal assays *Cladosporium sphaerospermum* (Penzig) CCIBt 491 and *C. cladosporioides* (Fresen) de Vries CCIBt 140 were cultured at the Instituto de Botânica, São Paulo, SP, Brazil. Ten microliters of a solution corresponding to 200 µg of fractions were dissolved in polarity-compatible solvents and applied on pre-coated TLC plates. For the hexane fraction, the TLC plates were developed with hexanes-ethyl acetate (9:1) and for the dichloromethane fraction with dichloromethane-methanol (9:1) as the mobile phase. After eluting, the plates were dried for complete removal of the solvents. The chromatograms were sprayed with a conidia suspension of *C. sphaerospermum* or *C. cladosporioides* (≥10^6^ conidia mL^−1^) in a glucose and salt solution and incubated for 48 h in a dark moistened chamber at 28 °C. A clear inhibition zone appeared against a dark background, indicating favorable sample activity. Nystatin (5 µg) was used as standard control [31].

## 5. Conclusions

In conclusion, the present study describes for the first time the occurrence of alkaloids in non-polar fractions of *Centroceras clavulatum*. Besides, the organic fractions obtained from *Centroceras clavulatum* showed interesting anti-protozoa results when submitted to *Tripanosoma cruzi* and antifungal potential against *Cladosporium sphaerospermum* and *C. cladosporioides*. Further chemical composition analyses showed that the environment can cause in some variations related to the structural metabolite diversity, such as when the Pakistan algae composition is compared with the Brazil species. So, it is clear that the environment and/or biodiversity can modify the species chemical constitution, showing us that the characteristics about one organism cannot be inferred for the same species from another ambient. 
